# Supplementary material for: Realizing a deep reinforcement learning agent for real-time quantum feedback
Source: Nat Commun. 2023 Nov 6;14:7138. doi: 10.1038/s41467-023-42901-3 (PMC10628214; doi:10.1038/s41467-023-42901-3)
Supplement: Supplementary file 1 — Supplementary Information [file 41467_2023_42901_MOESM1_ESM.pdf]

Supplementary Information Realizing a Deep Reinforcement Learning Agent for Real-Time Quantum Feedback

Kevin Reuer,[1,*] Jonas Landgraf,[2,3] Thomas Fösel,[2,3] James O'Sullivan,[1] Liberto Beltrán,[1] Abdulkadir Akin,[1] Graham J. Norris,[1] Ants Remm,[1] Michael Kerschbaum,[1] Jean-Claude Besse,[1] Florian Marquardt,[2,3] Andreas Wallraff,[1,4] and Christopher Eichler[1,†]

[1] *Department of Physics, ETH Zurich, CH-8093 Zurich, Switzerland*
[2] *Max Planck Institute for the Science of Light, Staudtstraße 2, 91058 Erlangen, Germany*
[3] *Physics Department, University of Erlangen-Nuremberg, Staudtstraße 5, 91058 Erlangen, Germany*
[4] *Quantum Center, ETH Zurich, CH-8093 Zurich, Switzerland*

(Dated: September 20, 2023)

SUPPLEMENTARY NOTE 1: EXPERIMENTAL SETUP AND DEVICE CALIBRATION

For the experiments, we use a transmon qubit coupled to a readout resonator on the chip shown in Fig. S1. The chip is mounted on the base temperature stage (20 mK) of a dilution refrigerator and housed inside three magnetic shields, two made from cryoperm, one from aluminum, see sketch of the experimental setup in Fig. S2. We apply microwave pulses to the chip via charge lines with 20 dB attenuation each on the 4 K, 100 mK and base temperature stage for signal conditioning [1]. To adjust the qubit frequency, we change the magnetic flux in its superconducting quantum interference device (SQUID) loop by generating currents in an inductively coupled flux line.

To readout the qubit, we generate a 256-ns-long microwave pulse at the readout frequency ω_{ro} with a microwave generator (MWG) and apply it to the readout resonator combined with its Purcell filter through the input line. The response of the resonator is then amplified by a traveling wave parametric amplifier (TWPA) with 20 dB gain, a high-electron mobility transistor (HEMT) and a room-temperatur amplifier (blue line in Fig. S2). We down-convert the readout signal to 250 MHz, using a local oscillator and an IQ mixer. For image rejection, we re-combine the I and Q channels of the IQ mixers using an IQ combiner, which adds a 90° phase shift to the Q channel. After further filtering and amplification, we digitize the signal with an analog-to-digital converter (ADC) and forward it to an FPGA. In the reinforcement learning approach for initializing the qubit (see main text), the agent on the FPGA selects an action, and if *flip* is chosen, triggers an arbitrary waveform generator (AWG) (dashed green line). The AWG then plays a pre-programmed derivative removal by adiabatic gate (DRAG) pulse [2], which is up-converted to the qubit frequency using a local oscillator and an IQ mixer. We then combine this conditional pulse with a periodically-triggered pulse channel (yellow box) used for preparation pulses and apply it to the qubit via a charge line (green line).

Using this setup, we achieve a feedback latency, defined

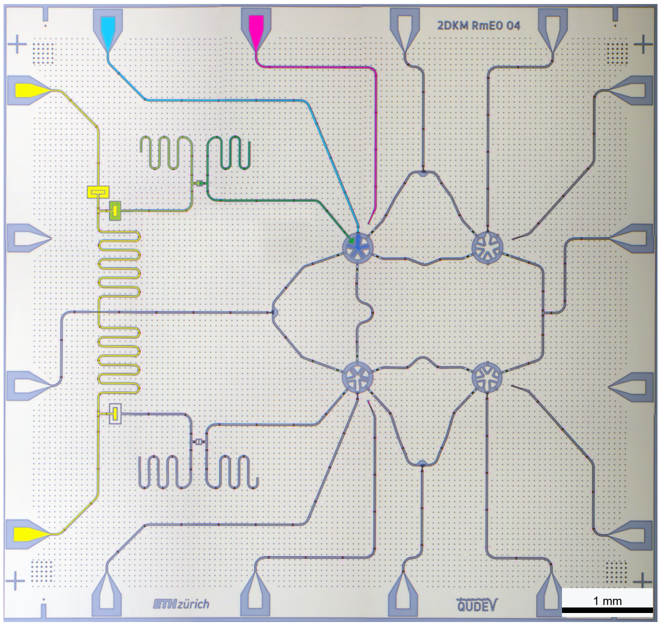

Figure S1. False color optical micrograph of the sample of the used transmon qubit (blue). Depicted are the readout resonator (green) coupled to the feed line (yellow) via a Purcell filter (light green). A flux line (cyan) and a charge line (pink) couple to the qubit. Uncolored parts of the chip are not used.

g-e frequency, $\omega_{ge}/2\pi$ [GHz]	6.524
e-f frequency, $\omega_{ef}/2\pi$ [GHz]	6.316
anharmonicity, $\alpha/2\pi$ [MHz]	-209
lifetime of $\lvert e\rangle$, $T_1^{(e)}$ [µs]	13
lifetime of $\lvert f\rangle$, $T_1^{(f)}$ [µs]	6
dephasing time of $\lvert e\rangle$, $T_2^{\star(e)}$ [µs]	2
dephasing time of $\lvert f\rangle$, $T_2^{\star(f)}$ [µs]	3
equilibrium excited state population, P_{therm} [%]	1.4
readout frequency, $\omega_{\mathrm{ro}}/2\pi$ [GHz]	7.259
dispersive shift, $\chi/2\pi$ [MHz]	10.4

Table S1. Measured device parameters.

* kevin.reuer@phys.ethz.ch
† christopher.eichler@fau.de

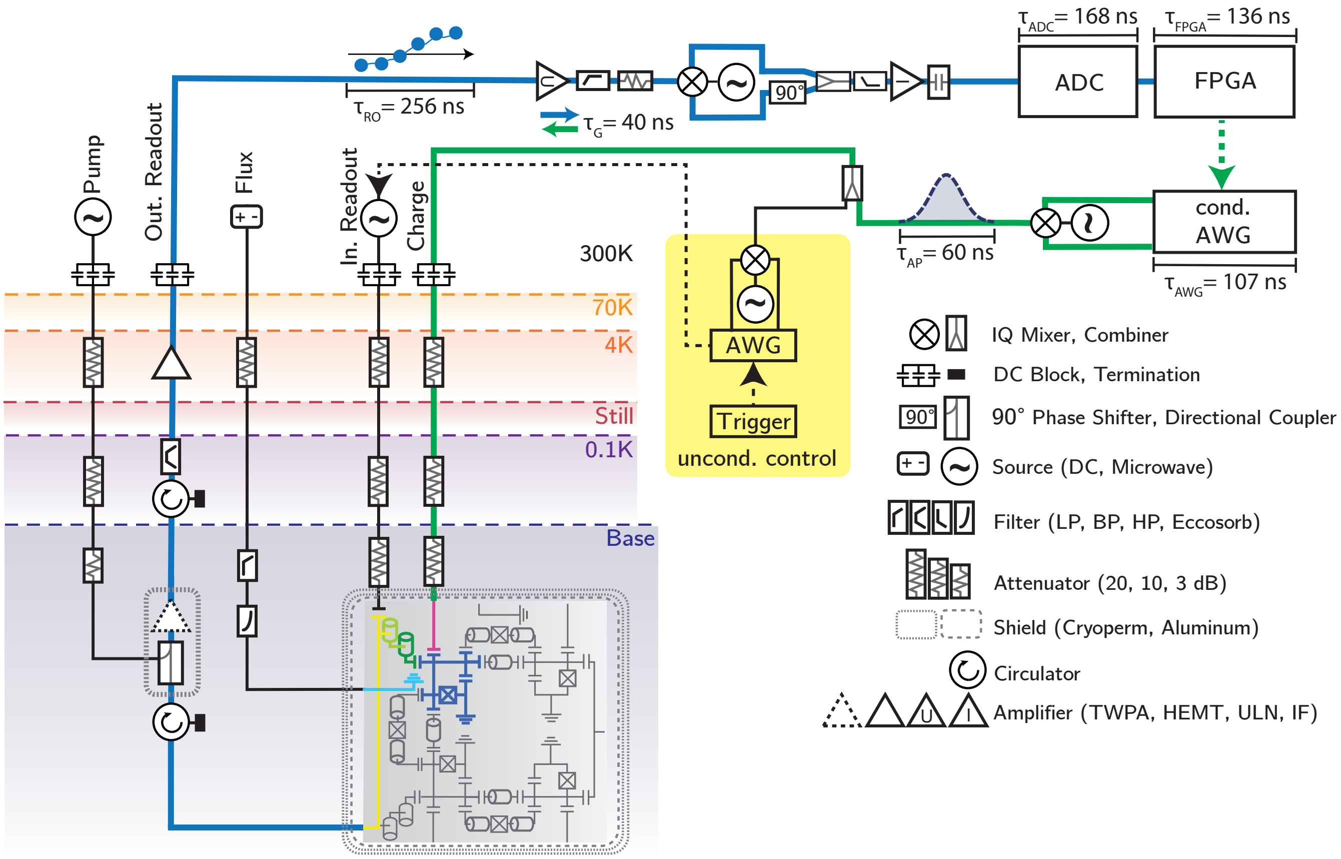

Figure S2. Experimental setup for operating the transmon qubit. For details, see text.

as the time between the end of the readout pulse and the start of the conditional π-pulse at the qubit, of $\tau_{\mathrm{EL,tot}} \approx 451$ ns. Main contributors to the latency are the ADC ($\tau_{\mathrm{ADC}} = 160$ ns, including the delay to generate a trigger for the AWG), the AWG ($\tau_{\mathrm{AWG}} = 107$ ns) and the FPGA ($\tau_{\mathrm{FPGA}} = 144$ ns). In addition, there is $\tau_{\mathrm{G}} \approx 40$ ns delay due the signal propagation from the room-temperature electronics to the sample and back through in total about 6 meters of coaxial cable. The latency of the neural network $\tau_{\mathrm{NN}} = 48$ ns is included in the FPGA latency τ_{FPGA}; inefficient signal pre-processing is responsible for 88 ns.

Considering the readout pulse duration $\tau_{\mathrm{RO}} = 256$ ns and the duration of the $\pi-$pulse flipping the qubit $\tau_{\mathrm{AP}} = 60$ ns, we obtain a minimum cycle duration $\tau_{\mathrm{cycle,min}} = \tau_{\mathrm{RO}} + \tau_{\mathrm{AP}} + \tau_{\mathrm{EL,tot}} \approx 767$ ns. When including a *gf-flip*, implemented as a π-pulse on the $|f\rangle$-$|e\rangle$ manifold, followed by a π-pulse on the $|e\rangle$-$|g\rangle$ manifold ($\tau_{\mathrm{AP}} = 112$ ns), we find $\tau_{\mathrm{cycle,min}} = 819$ ns. For the presented experiments, we have chosen a cycle time of 856 ns for both cases, leaving room for further optimization of the cycle time by about 40 ns in future experiments.

We measure the basic device parameters presented in Table S1 by performing single- and two-tone spectroscopy, as well as Rabi, Ramsey and coherence measurements for the ground state to excited state and excited to second excited state transitions, as presented in Ref. [3].

SUPPLEMENTARY NOTE 2: READOUT CHARACTERIZATION AND POPULATION EXTRACTION

To characterize the readout detection chain, we measure the dephasing $\beta = |\rho_{01,\mathrm{on}}(T)|/|\rho_{01,\mathrm{off}}(T)|$ induced by a readout pulse of length T, where $\rho_{01,\mathrm{on}}(t)$ is time dependent off-diagonal element of the qubit's density matrix with the readout pulse present, while $\rho_{01,\mathrm{off}}(t)$ is for without the readout pulse. We then compare β to the signal-to-noise ratio (SNR) of the processed readout signal [4], see Fig. S4(a). As, for a given dephasing β, $\sqrt{4\beta}$ is the maximum possible SNR, we define the quantum efficiency η as $\eta = \mathrm{SNR}^2/4\beta$. We measure β in a Ramsey-like experiment, applying a readout pulse of varying amplitude in between the two $\pi/2$ pulses. For these amplitudes, we then also evaluate the SNR of the measurement signal, by preparing the qubit in $|g\rangle$ and $|e\rangle$, creating a histogram of the integrated signal V and fitting a bimodal Gaussian distribution $a_g\mathcal{N}(\mu_g, \sigma_g^2) + a_e\mathcal{N}(\mu_e, \sigma_e^2)$, with means μ_g, μ_e, variances σ_g^2, σ_e^2 and amplitudes a_g,a_e to V. The SNR is then defined as $\mathrm{SNR}^2 = |\mu_g - \mu_e|^2/\sigma_g^2$. We observe, as expected, a linear dependence between SNR^2 and 4β,

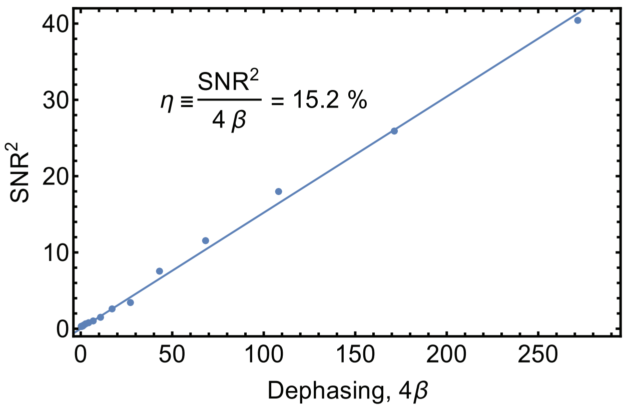

Figure S3. Squared signal-to-noise ratio SNR^2 vs four times the measurement induced dephasing 4β for different readout powers. Line is a linear fit to the data, η is extracted from the fit.

see Fig. S3, and we obtain the quantum efficiency of $\eta = 15.2\%$ from a linear fit to the data, likely due to losses before the TWPA and added noise by amplifiers after the TWPA, whose gain was not large enough to overcome other noise sources.

Furthermore, we evaluate the performance of the readout in different regimes by extracting the readout infidelity $1 - \mathcal{F}$. For this purpose, we prepare the qubit in $|g\rangle$, $|e\rangle$ (and second excited $|f\rangle$) states, after heralding the ground state with a pre-selection readout pulse, and fit a bimodal (trimodal) Gaussian distribution to the combined histogram of both (all) prepared states, see Fig. S4. For two-level-readout, we define a threshold $t = (\mu_g + \mu_e)/2$ and assign shots with $V < t$ to $|g\rangle$ and with $V > t$ to $|e\rangle$, see Fig. S4(a,b). By counting the missassigned shots, we extract $P(g|e)$, the probability to assign a prepared excited state to $|g\rangle$, and $P(e|g)$ the probability to assign a prepared ground state to $|e\rangle$, and obtain the readout infidelity of $1 - \mathcal{F} = \frac{1}{2}(P(g|e) + P(e|g)) = 1.95$ % for strong and 13.9 % for weak measurements. The strong measurements are limited by the decay of the excited state into the ground state during the 256 ns-long readout pulse, while overlap errors dominate in the weak measurement case. For three-level readout, we define three assignment regions based on the fitted Gaussian distributions and obtain an infidelity of $1 - \mathcal{F} = 11.3$ %, see Fig. S4(c). We note that the readout was optimized for two-level readout, resulting in the comparatively large error [5, 6] when choosing to distinguish between all three states.

These finite readout infidelities $1 - \mathcal{F}$ will lead to errors in the extraction of the initialization error $1 - P_g$ when using thresholding. Therefore we use a a different method: We first obtain the means μ_g and μ_e and variances σ_g^2 and σ_e^2 from a fit of a bimodal Gaussian distribution to the histogram of the initial equilibrium state. For the weak measurement case, we facilitate the fitting by assuming $\sigma_g^2 = \sigma_e^2$, as this is expected for low readout powers. In the three-level case, we fit a two-dimensional tri-modal Gaussian distribution $a_g\mathcal{N}(\mu_g, \Sigma_g) + a_e\mathcal{N}(\mu_e, \Sigma_e) + a_f\mathcal{N}(\mu_f, \Sigma_f)$ to the two-dimensional histogram of V and W of the initial equilibrium state, and obtain means μ_g, μ_e and μ_f and covariance matrices Σ_g, Σ_e and Σ_f. In a second step, we then fit the amplitudes a_g and a_e (a_f) to the histogram of V (and W) from the verification measurement, using the previously obtained means and variances (covariance matrices). The extracted populations are then given by the amplitude ratios $P_g = a_g/(a_g + a_e)$ and $P_e = a_e/(a_g + a_e)$ ($P_g = a_g/(a_g + a_e + a_f)$, $P_e = a_e/(a_g + a_e + a_g)$ and $P_f = a_e/(a_g + a_e + a_f)$). We note that the binning of the shots in the histogram leads to Poissonian noise, making standard least squares fitting procedures inaccurate. Instead we use a maximum likelihood procedure as described in Ref. [7] to fit the bi-/tri-modal Gaussian distributions.

We use the described techniques to analyze the bimodal Gaussian distribution in Fig. 4(e). We deduce that the agent's performance is limited mostly by rethermalization as the integrated signal in the termination cycle (green diamonds) has only very few counts above the state discrimination threshold, while the number of such instances rises to about 0.18% in the verification measurement, indicating transitions into the excited state occuring between the two cycles. Compared to the equilibrium state (blue circles) the excited state fraction is reduced by about a factor 10 by using the reinforcement learning initialization scheme.

SUPPLEMENTARY NOTE 3: STATE DISCRIMINATION WITH NEURAL NETWORKS

Since qubit state initialization relies on the distinguishability between the two states given an observation $\mathbf{s}$, we also study the ability of the neural network to accomplish this task. We compare its performance with the one of a standard classifier, which integrates $\mathbf{s}$ with a set of optimal filter coefficients $\mathbf{w_s}$ to obtain $V = \mathbf{s} \cdot \mathbf{w_s}$ and assigns a state by thresholding V [8, 9]. To train the neural network in assigning the correct state, we use supervised learning [10–12] on a labeled data set consisting of 8212 individual time-traces in which we prepare ground and excited states after heralding an initial ground state with a pre-selection readout. The performance of the neural network classifier is evaluated based on an independent validation data set, which was interleaved with the training data set.

The two example time-traces for prepared $|g\rangle$ and $|e\rangle$ states (dashed blue and orange lines in Fig. S5(a)) become distinguishable on a timescale of about 50 ns. The fluctuations around their respective average response $\langle \mathbf{s}_g \rangle$ and $\langle \mathbf{s}_e \rangle$ (solid orange and blue lines) are dominated by Gaussian noise added during the amplification process. In addition, there are few instances in which the time-dependent signal suddenly changes its amplitude (black trace in Fig. S5(a)), indicating possible state transition events during the measurement mostly due to decay from $|e\rangle$ to $|g\rangle$.

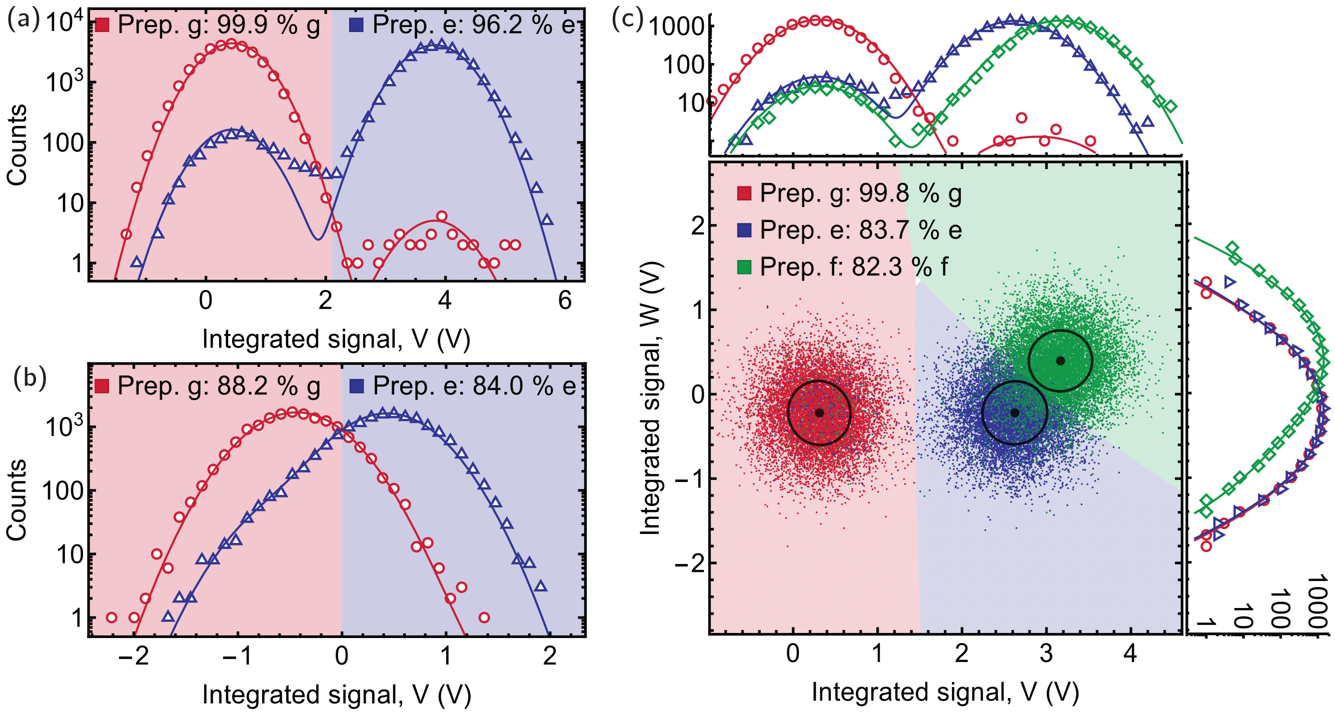

Figure S4. (a,b) Histogram of the integrated readout signal V, when preparing a ground (g, red) and excited state (e, dark blue) for (a) strong and (b) weak measurements, after heralding the ground state with a pre-selection readout pulse. Lines are a bimodal Gaussian fit to the data. (c) Two-dimensional histogram of the integrated readout signals V and W (see main text), when preparing a ground (g, red), excited (e, dark blue) or second excited (f, green) state after heralding the ground state with a pre-selection readout pulse. Black points and circles indicate the fitted means and standard deviation ellipses. Marginal distributions with the corresponding fits are shown in the top and right subpanel. For each panel, all prepared states are fitted with the same means and variances, but different amplitudes. Assignment regions are shown as background colors.

For short measurement times τ up to 200 ns the readout infidelity $1-\mathcal{F} = \frac{1}{2}\left(P(g|e) + P(e|g)\right)$, where $P(i|j)$ is the fraction of states prepared in state $|j\rangle$ and assigned to $|i\rangle$, decreases for both classifiers when increasing τ, see Fig. S5(b). The neural network's performance matches the readout fidelity of the standard classifier, which is known to be optimal for integration times much shorter than the qubit lifetime $\tau \ll T_1$. For longer measurement times τ, the readout infidelity of the standard classifier starts to increase because of state transitions during the measurement, which the linear filtering technique cannot resolve. In contrast, the neural network's performance further improves up until $\tau \approx 300$ ns and stays constant afterwards as the neural network is able to detect such state transition events. Thus the neural network outperforms the standard classifier in this regime. For example, the neural network classifier correctly assigned the time-trace shown in black in Fig. S5(a), while it was misclassified by the standard classifier. We note that for the two outliers in the performance of the neural network (around $\tau = 0.75$ µs and $\tau = 1.8$ µs in Fig. S5(b)) the training algorithm most likely converged to non-optimal network parameters.

SUPPLEMENTARY NOTE 4: EXPERIMENTAL REINFORCEMENT LEARNING

AlgorithmS1 Training algorithm

Require: Initial network parameters θ and ζ of the policy and critic network π_θ and V_ζ
for training step=1,2,...,N_{steps} **do**
  Transfer θ to the FPGA
  Record episodes in the quantum system
  Transfer episodes to the PC
  Evaluate the critic network V_ζ
  Calculate the rewards (see Eq. (1))
  Update θ and ζ with PPO [13, 14]
end for

To train the network, we perform 500 training steps, i.e. 500 updates of the neural network parameters θ, in around 8 min, starting from a random policy. During each training step, the FPGA records initialization episodes with a cycle time of 856 ns and a repetition rate of 10 KHz until 1000 measurements have been carried out. The measurement outcomes and the chosen actions are transferred to a personal computer (PC) in around 0.6 s. The PC updates the parameters θ of the policy network π_θ within

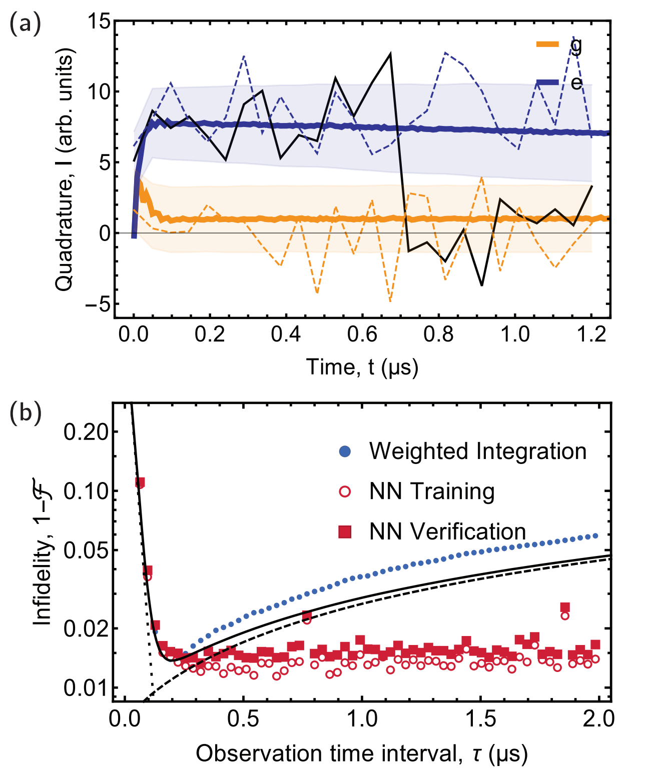

Figure S5. State discrimination via neural network. (a) Measured average (solid lines, $\pm\sigma$ standard deviation shaded) and single-shot (dotted) quadrature I (single-shot down-sampled with a six point boxcar filter) for the qubit being in the ground (orange) or the excited (dark blue) state, as well as a single-shot during a potential decay event (black). (b) Readout infidelity $1-\mathcal{F}$ with respect to the prepared state vs observation time τ, when assigning the states with the standard classifier (blue dots) and with a trained neural network classifier for a verification data set (red squares) and a training data set (red circles), respectively. Simulated readout infidelity of the standard classifier (solid black line), considering overlap errors (dotted) and errors due to decoherence (dashed).

0.1 s, and the updated parameters θ are transferred back to the FPGA in around 0.3 s.

The only accessible information during the initialization procedure is the measurement results during the feedback loop and the verification measurements; thus, the reward function can only be based on them. We choose the reward r^t at time step $t \in \{1, ..., n-1\}$ as

$$r^t = \frac{V_{t+1} - V_t}{V_g - V_e} - \lambda \quad (1)$$

with the integrated observation $V_t = \mathbf{w}_s \mathbf{s}^t$ of the tth cycle V_t and a control parameter λ. V_g and V_e are the average integrated observation if the qubit is prepared in the ground or excited state. For the final time step the reward r^n is defined as

$$r^n = \frac{V_{\text{ver}} - V_n}{V_g - V_e} - \lambda \quad (2)$$

Hyperparameter	Value
Adam parameter η	5×10^{-4}
Adam parameter β_1	0.98
Adam parameter β_2	0.999
Discount rate γ	0.92
Entropy coefficient	0.01
Cliprange	0.04
λ of the generalized advantage estimation	0.98
Number of training minibatches per update	1
Number of epochs for surrogate optimization	8
Maximum value for gradient clipping	∞

Table S2. Hyperparameters used for training, for definition of the hyperparameters see [13, 14]

using the integrated observation in the final verification measurement $V_{\text{ver}} = \mathbf{w}_s \mathbf{s}^{\text{ver}}$. The integration weights $\mathbf{w}_s$ are chosen to maximize signal-to-noise ratio under Gaussian noise [5, 8–10]. The noise in the readout signal originates from Gaussian noise of vacuum fluctuations, losses in the readout line, and added noise of amplifiers [9]. Therefore, $V_{t+1} - V_t$ is a good indicator for the progress of the initialization compared to the previous round and gives direct information if the action a_t resulted in a quantum state closer to the target state. The parameter λ penalizes every action and thus controls the trade-off between average episode length and initialization fidelity.

The network parameters θ are modified in every update step to maximize the averaged cumulative reward $\langle R \rangle$, defined as

$$\langle R \rangle = \left\langle \sum_{t=1}^{n} r^t \right\rangle \quad (3)$$

where $\langle \cdot \rangle$ denotes the average over all possible episodes. With our chosen reward function, the average cumulative reward equals

$$\langle R \rangle = \frac{\langle V_{\text{ver}} \rangle - V_e}{V_g - V_e} - \lambda \langle n \rangle + \text{const.} \quad (4)$$

The first term approximates the initialization fidelity, the second one penalizes long episodes and the constant is independent of the agent's policy.

For the training step we use the Proximal Policy Optimization (PPO) algorithm [13] from the Python library Stable Baselines [14]. We choose PPO as a state-of-the-art algorithm widely used in the machine learning community. In addition to the policy network on the FPGA, the PPO algorithm makes use of a second network, the so-called critic network V_ζ with its parameters ζ. Based on the current observation, the critic estimates the expected future cumulative reward given the current policy π_θ. By comparing the cumulative reward for each observation collected on the FPGA to the expectation of the critic, the PPO algorithm identifies action sequences that perform better than expected and modifies the agent's policy such that the agent is more likely to select these action sequences. The critic is only required for the update step

while it is not required for the decision-making process. Therefore, the critic network is only running on the PC. We implement the critic network as a feedforward network with two hidden layers with 64 neurons per layer. All additional hyperparameters of the PPO algorithm are listed in S2.

The whole training loop described above is summarized in Algorithm S1.

SUPPLEMENTARY NOTE 5: LOW-LATENCY NEURAL NETWORK IMPLEMENTED ON THE FPGA

For our FPGA implementation we introduce a novel network architecture, which aims to keep latencies at a minimum, see Fig. 2(b). First, we implement the agent as a feedforward neural network [15] on the FPGA, rather than a more resource-demanding recurrent neural network, like a long short-term memory network (LSTM) [16]. Second, we process information from previous cycles in a two-layer *pre-processing network* before the start of the current cycle, thus not contributing to the latency. Third, and most importantly, we implement a novel *low-latency network* architecture, in which new measurement data is processed as soon as it becomes available. More specifically, we sequentially feed elements I_k^j, Q_k^j of the digitized time trace $\mathbf{s}^j = (\mathbf{I}^j, \mathbf{Q}^j)$ into each layer of the neural network concurrent with its evaluation, see Fig. 2(b). As a result, only the execution of the last layer contributes to the total latency while all other layers are evaluated in parallel with the data acquisition. We note that data points fed into later layers are processed less than data points fed into earlier layers. In simulations, however, this did not affect the performance of the reinforcement learning agent. For the experiments presented in the following, we use a network with 7 hidden layers and 12 neurons per layer. The output layer has only three neurons, corresponding to the three actions. However, as the exact neural network structure may in general depend on the properties of the specific quantum system and the agent's task, the width and depth of the neural network are adjustable parameters in our FPGA design. In the following, we discuss design considerations of the neural network architecture to reach this goal by making optimal use of the available FPGA resources.

Our network is a feedforward network and consists of multiple dense layers, each of which transforms the values of N input neurons $\mathbf{y}^{(\text{in})}$ into the values of M output neurons $\mathbf{y}^{(\text{out})}$ according to

$$y_j^{(\text{out})} = f\left(\sum_{k=0}^{N-1} w_{jk} y_k^{(\text{in})} + b_j\right) \tag{5}$$

with $j \in \{0, ..., M-1\}$. Here, f is the nonlinear activation function, w the $M \times N$-dimensional kernel matrix and $\mathbf{b}$ the M-dimensional bias vector (where w and $\mathbf{b}$ are different for different layers).

To compute the values of all $y_j^{(\text{out})}$ in parallel and with minimum latency on the FPGA we multiply all $y_k^{(\text{in})}$ with their respective weights w_{jk} within one clock cycle of duration $\tau_{\text{clock}} = 8\,\text{ns}$ and then add them up sequentially in subsequent clock cycles, see S6. Since two subsequent additions are performed within one clock cycle, the number of summands gets reduced in each clock cycle by at most a factor $2^2 = 4$, such that the total number of clock cycles required to perform the summation is $\lceil \log_4(N+1) \rceil$, where the "+1" accounts for the bias b_j. We evaluate the nonlinear activation function f chosen to be the Rectified Linear Unit (ReLU) function in the last clock cycle. Therefore, the execution time τ_{dense} of a single dense layer is given by

$$\tau_{\text{dense}} = \tau_{\text{clock}} \left(1 + \lceil \log_4(N+1) \rceil\right), \tag{6}$$

In our specific experiment, we choose $N = 20$ for each layer of the low-latency network resulting in an execution time of 32 ns per layer.

We equip our network with a memory of the past by providing it in each cycle t with the readout signals s^j and actions a^j from l previous rounds. As this information is already available after the previous action was selected, s^j and a^j from l previous rounds are evaluated in a *pre-processing network* (see Fig. 3) while the agent is waiting to receive the most recent readout signal $\mathbf{s}^t$ of the current cycle. Thus, no additional latency is introduced to the feedback loop. However, the evaluation needs to be completed when the most recent readout signal is received. Since evaluating the pre-processing network takes more time when taking more previous rounds l into account, we chose to limit l to two in our implementation. To overcome this limitation in future work, we could parallelize the evaluation of the pre-processing network, enabling it to process information from up to 6 previous cycles. To reduce the amount of data to be processed, we apply a 32-point boxcar filter to the previous measurement results before feeding them into the network. Each of the previous actions is expressed by a three-bit string. A fourth bit is added if the *gf*-flip action is considered. The preprocessing network consists of two layers with 12 neurons per layer.

We start to evaluate the network as soon as the first element of the signal arrives. The first layer processes this information together with the output of the preprocessing network. Layer by layer, the most recent measurement data is fed into the network until the whole signal is processed.

The measurement signal, recorded with a time resolution of 1 ns, is down-sampled with an eight-point boxcar filter, introducing a latency of 16 ns. We feed four elements of the in-phase and out-of-phase component of the down-sampled signal and the output of 12 neurons from the previous layer into the subsequent layer resulting in an input size of $N = 20$.

The last layer has one output neuron per action and each neuron value encodes the probability of choosing its corresponding action. In order to sample an action, we

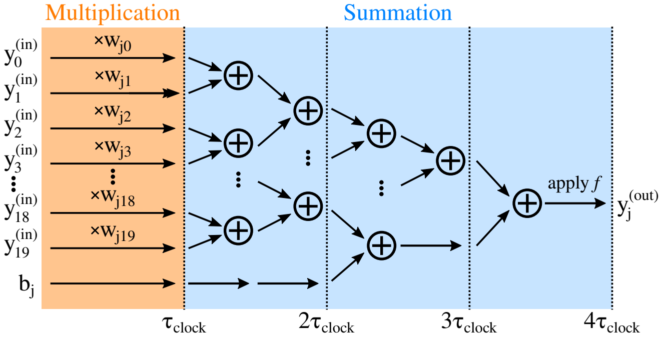

Figure S6. Evaluation for $N = 20$ input neurons on the FPGA. All output neurons are evaluated in parallel. In the first clock cycle, the inputs are multiplied with their respective weights. In the following cycles, these products and the bias are summed pairwise. Two summations are performed per clock cycle.

use the Gumbel-max trick [17] which does not introduce any additional latencies.

The network execution adds a latency of 48 ns, where 16 ns result from the eight-point boxcar filter and 32 ns from the execution of the last layer.

[1] S. Krinner, S. Storz, P. Kurpiers, P. Magnard, J. Heinsoo, R. Keller, J. Lütolf, C. Eichler, and A. Wallraff, Engineering cryogenic setups for 100-qubit scale superconducting circuit systems, EPJ Quantum Technology **6**, 2 (2019).

[2] F. Motzoi, J. M. Gambetta, P. Rebentrost, and F. K. Wilhelm, Simple pulses for elimination of leakage in weakly nonlinear qubits, Phys. Rev. Lett. **103**, 110501 (2009).

[3] J.-C. Besse, K. Reuer, M. C. Collodo, A. Wulff, L. Wernli, A. Copetudo, D. Malz, P. Magnard, A. Akin, M. Gabureac, G. J. Norris, J. I. Cirac, A. Wallraff, and C. Eichler, Realizing a deterministic source of multipartite-entangled photonic qubits, Nat. Commun. **11**, 4877 (2020).

[4] C. C. Bultink, B. Tarasinski, N. Haandbæk, S. Poletto, N. Haider, D. J. Michalak, A. Bruno, and L. DiCarlo, General method for extracting the quantum efficiency of dispersive qubit readout in circuit qed, Appl. Phys. Lett. **112**, 092601 (2018).

[5] P. Magnard, P. Kurpiers, B. Royer, T. Walter, J.-C. Besse, S. Gasparinetti, M. Pechal, J. Heinsoo, S. Storz, A. Blais, and A. Wallraff, Fast and unconditional all-microwave reset of a superconducting qubit, Phys. Rev. Lett. **121**, 060502 (2018).

[6] S. Krinner, N. Lacroix, A. Remm, A. D. Paolo, E. Genois, C. Leroux, C. Hellings, S. Lazar, F. Swiadek, J. Herrmann, G. J. Norris, C. K. Andersen, M. Müller, A. Blais, C. Eichler, and A. Wallraff, Realizing repeated quantum error correction in a distance-three surface code, Nature **605**, 669 (2022).

[7] T. A. Laurence and B. A. Chromy, Efficient maximum likelihood estimator fitting of histograms, Nature Methods **7**, 338 (2010).

[8] J. Gambetta, W. A. Braff, A. Wallraff, S. M. Girvin, and R. J. Schoelkopf, Protocols for optimal readout of qubits using a continuous quantum nondemolition measurement, Phys. Rev. A **76**, 012325 (2007).

[9] T. Walter, P. Kurpiers, S. Gasparinetti, P. Magnard, A. Potočnik, Y. Salathé, M. Pechal, M. Mondal, M. Oppliger, C. Eichler, and A. Wallraff, Rapid, high-fidelity, single-shot dispersive readout of superconducting qubits, Phys. Rev. Appl. **7**, 054020 (2017).

[10] Magesan, E., Gambetta, J. M., Córcoles, A. D., and Chow, J. M., Machine learning for discriminating quantum measurement trajectories and improving readout, Phys. Rev. Lett. **114**, 200501 (2015).

[11] E. Flurin, L. S. Martin, S. Hacohen-Gourgy, and I. Siddiqi, Using a recurrent neural network to reconstruct quantum dynamics of a superconducting qubit from physical observations, Phys. Rev. X **10**, 011006 (2020).

[12] B. Lienhard, A. Vepsäläinen, L. C. G. Govia, C. R. Hoffer, J. Y. Qiu, D. Ristè, M. Ware, D. Kim, R. Winik, A. Melville, B. Niedzielski, J. Yoder, G. J. Ribeill, T. A. Ohki, H. K. Krovi, T. P. Orlando, S. Gustavsson, and W. D. Oliver, Deep-neural-network discrimination of multiplexed superconducting-qubit states, Phys. Rev. Applied **17**, 014024 (2022).

[13] J. Schulman, F. Wolski, P. Dhariwal, A. Radford, and O. Klimov, Proximal Policy Optimization Algorithms, arXiv:1707.06347 [cs] (2017).

[14] A. Hill, A. Raffin, M. Ernestus, A. Gleave, A. Kanervisto, R. Traore, P. Dhariwal, C. Hesse, O. Klimov, A. Nichol, M. Plappert, A. Radford, J. Schulman, S. Sidor, and Y. Wu, Stable baselines (2018).

[15] I. Goodfellow, Y. Bengio, and A. Courville, *Deep Learning* (MIT Press, 2016).

[16] S. Hochreiter and J. Schmidhuber, Long short-term memory, Neural Computation **9**, 1735 (1997).

[17] I. A. M. Huijben, W. Kool, M. B. Paulus, and R. J. G. van Sloun, A Review of the Gumbel-max Trick and its Extensions for Discrete Stochasticity in Machine Learning, arXiv:2110.01515 [cs, stat] (2021).
